# Minimally invasive autopsies for the investigation of pulmonary pathology of COVID-19—experiences of a longitudinal series of 92 patients

**DOI:** 10.1007/s00428-023-03622-6

**Published:** 2023-09-01

**Authors:** Petar Noack, Claudia Grosse, Jacob Bodingbauer, Marion Almeder, Sylvia Lohfink-Schumm, Helmut J.F. Salzer, Jens Meier, Bernd Lamprecht, Clemens A. Schmitt, Rupert Langer

**Affiliations:** 1 Institute of Clinical Pathology, Kepler University Hospital, Krankenhausstr. 9, 4021 Linz, Austria; 2 Medical Faculty, Johannes Kepler University, Linz, Austria; 3 Division of Infectious Diseases and Tropical Medicine, Department of Pulmonary Medicine, Kepler University Hospital, Linz, Austria; 4 Ignaz-Semmelweis-Institute, Interuniversity Institute for Infection Research, Vienna, Austria; 5 Department of Anesthesiology and Intensive Care Medicine, Kepler University Hospital, Linz, Austria; 6 Department of Pulmonary Medicine, Kepler University Hospital, Linz, Austria; 7 Department of Hematology and Medical Oncology, Kepler University Hospital, Linz, Austria

**Keywords:** Minimally invasive autopsy, Pulmonary pathology, Diffuse alveolar damage, COVID-19

## Abstract

Minimally invasive autopsies (MIAs) allow the collection of tissue samples for diagnostic and research purposes in special situations, e.g., when there is a high risk of infection which is the case in the context of COVID-19 or restrictions due to legal or personal reasons. We performed MIA to analyze lung tissue from 92 COVID-19 patients (mean age 78 years; range 48–98; 35 women, 57 men), representing 44% of all patients who died from the disease between October 2020 and April 2021. An intercostal approach was used with removal of a 5-cm rib section followed by manual collection of four lung tissue samples (5–8 cm in size). Diffuse alveolar damage (DAD) was found in 89 (97%) patients at various stages. Exudative DAD (eDAD) predominated in 18 (20%) patients, proliferative DAD (pDAD) in 43 (47%) patients, and mixed DAD (mDAD) in 31 (34%) patients. There were no significant differences in the predominant DAD pattern between tissue samples from the same patient. Additional purulent components were present in 46 (50%) cases. Fungi were detected in 11 (12%) patients. The pDAD pattern was associated with longer hospital stay including intensive care unit (*p*=0.026 and *p*<0.001) and younger age (*p*=0.019). Positive bronchoalveolar lavage and blood cultures were observed more frequently in pDAD patterns (*p*<0.001; *p*=0.018). In contrast, there was no significant association between intravital positive microbiological results and superimposed bronchopneumonia or fungal infection at autopsy. Having demonstrated the characteristic lung changes in a large longitudinal autopsy series, we conclude that the presented MIA approach can be considered a reliable and safe method for performing post mortem lung diagnostics in COVID-19 and other high-risk situations. The lack of correlation between histological changes indicative of bacterial or fungal superinfection and microbiology could have clinical implications for disease and treatment surveillance.

## Introduction

Soon after the beginning of the SARS-COV2 pandemic, studies on autopsy-derived tissues from persons who died from coronavirus-induced disease (COVID-19) provided valuable contributions to the understanding of this disease. It was demonstrated that the main lung finding was diffuse alveolar damage (DAD) in different stages depending on the duration and the severity of the disease [1–4]. Subsequently, these initial findings of severe pulmonary changes were confirmed in several case series [4–8] and summarized in review articles and meta-analyses [1, 9–13].

Many original data published so far, though, are often based on relatively small case series with currently only one published single-center study of 100 cases [14]. On the one hand, this is due to the legal background regarding post mortem diagnostics and the unfortunately rather small role of autopsies in modern medicine in most countries of the world [15] which resulted in remarkably low autopsy rates of deceased COVID-19 patients compared with the number of deaths. On the other hand, safety issues that have to be taken into account when performing autopsies on patients with this highly transmissible disease are also very complex even under vaccination protection of staff [1, 16, 17]. As a consequence, large-scale single-center tissue-based COVID-19 studies are still lacking [6, 18].

Minimally invasive autopsy (MIA) techniques have been successfully introduced in post mortem diagnostics [19]. There is a broad spectrum of techniques ranging from laparoscopic and thoracoscopic procedures to percutaneous biopsies with or without assistance through imaging techniques. MIA is considered very helpful, e.g., in the setting of infectious diseases or in case of reservations of relatives offering the possibility to obtain tissue samples for both diagnostic and research purposes with limited risk of infection and without the need of opening cavities in particular situations [20–24].

In the following, we report our experience using a MIA approach for the investigation of lung tissue of a large case series of COVID-19 patients who died between October 2020 and April 2021 (the so-called second wave in Central Europe [25]). We chose a simple intercostal MIA technique for the collection and analysis of lung tissue. Four tissue samples measuring 5–8 cm were obtained from each patient and evaluated by histology using a standardized protocol. The findings were then set in context to clinical, epidemiological, and microbiological data.

## Material and methods

### Minimally invasive autopsy technique for the investigation of lung tissue

**Fig. 1:**
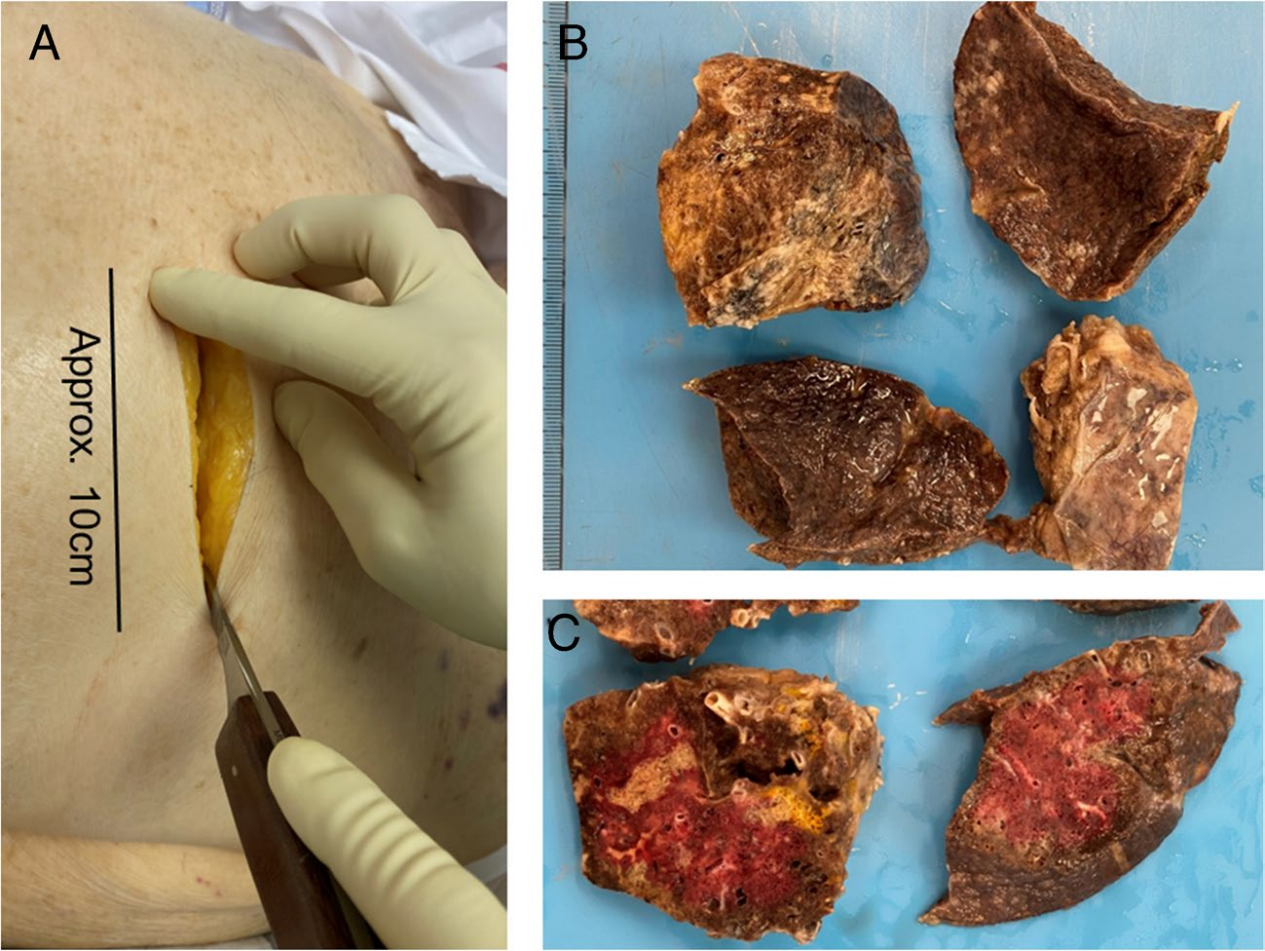
**A** Transcostal approach; **B**, **C** macroscopy of obtained tissue samples, allowing macroscopical tissue evaluation

At the beginning of the “second wave” of the COVID-19 pandemic in October 2020 [25], in view of the overwhelming number of COVID-19 patients deceased at the Kepler University Hospital Linz, Austria, and associated work load in the autopsy unit, a simple method for minimally invasive surgical sampling of lung tissue at the Institute of Clinical Pathology and Molecular Pathology of the Kepler University Hospital and the Johannes Kepler University Linz, Austria, was developed. This technique allows generous preservation of lung tissue from COVID-19 deceased patients with low risk of infection and acceptable time expenditure. The interventions were performed under general safety measures [16]. During the procedure, the bodies remained within the double body bags that were used for deceased people according to the instruction of the hospital. MIA was performed using an intercostal approach with a 10-cm long skin opening in between the 4th and the 5th, the 5th and the 6th, or the 6th and the 7th rib. Next, 5-cm pieces of the ribs were removed using rib shears to create a window for better handling. This was followed by further dissection of the pleura and manual collection of four samples of lung tissue (5–8 cm size) from the upper and lower lobes of both lungs of each patient (Fig. 1).

Taken into account dropouts due to logistical factors, such as increased routine workload in the autopsy department of the institute after weekends which comprised extensive paperwork and handling over the bodies of all deceased patients of the hospital to the undertakers, we were able to collect lung tissue samples from 92 patients diagnosed with SARS-COV2 infection prior to death. This represents almost half (44%) of all 212 patients who died of COVID-19 at the Kepler University Hospital between October 2020 and April 2021.

### Histological evaluation

After the MIA procedure, the samples fixed in formalin for at least 48 h which was then followed by grossing, taking one representative tissue block per sample and routine histopathologic work up. The samples were stained with hematoxylin and eosin. The characterization of lung damage and inflammatory infiltrates, as well as additional findings, was performed. Elastica van Gieson, PAS, and Grocott (GMS) stainings were additionally performed on request depending on the findings firstly obtained by hematoxylin and eosin staining.

The following parameters were then evaluated in a standardized manner for each slide and according to previously published studies [26, 27].

Presence and semiquantitative estimation (grades 0–3) of the degree of diffuse alveolar damage, exudative phase (eDAD), are characterized by the presence of hyaline membranes, edema, and congestion; presence and semiquantitative estimation (grade 0–3) of the degree of DAD, proliferative phase (pDAD), are defined by fibroblastic proliferations in the interstitial and alveolar spaces. Fully developed late-stage fibrosis, with structural remodeling (honeycomb lungs), was not observed.

Superimposed acute bronchopneumonia is defined by the presence of more than few granulocytes [27] and the severity graded into 0–3, presence or absence of microthrombi, presence or absence of squamous metaplasia, presence or absence of fungal organisms, and additional pathologic findings.

For eDAD and pDAD, a total score was then calculated by adding the single scores of the slides; scores 0–4 were categorized as low, scores 5–8 as medium, and 9–12 as high level. Similarly, superimposed acute bronchopneumonia was categorized into absent, low (scores 1–4), and high level (scores 5–12). Presence of fungi was categorized into presence and absence in at least one slide. Presence of microthrombi and squamous metaplasia was categorized as absent (in none of the slides), low (in one slide), and high (in more than one slide).

### Clinical data

Clinical characteristics were obtained from the electronical patient records of the clinical information system. They included the diagnostic CT values from the nasopharyngeal swabs, duration of hospitalization from the day of submission to the day of death, time at the intensive care unit if appropriate, information about oxygenation (normal oxygen supply, non-invasive oxygenation, invasive oxygenation with or without extracorporeal membranous oxygenation (ECMO)), comorbidities (separated into cardiovascular including arterial hypertension; pulmonary; diabetes mellitus; immunosuppression; renal and hepatic comorbidities), therapy and microbiological findings from blood cultures and bronchioalveolar lavages.

### Statistics

Descriptive results of histological and clinical data are reported as absolute and relative frequencies for nominal variables and by summary statistics (median, maximum, and minimum) for metric variables with non-normal distribution. Correlations between metric variables are quantified by Spearman’s correlation coefficients. For the comparison of nominal values between groups, non-parametric tests were applied (*X*^2^ or Fisher’s exact tests for nominal, Mann-Whitney *U* Test for ordinal variables). For all calculations, the IBM SPSS Statistics program (version 28.0) was used.

### Ethical considerations

The usage of autopsy derived tissue samples including controls for the assessment of COVID-19 specific findings has been approved by the Ethics Commission of the Johannes Kepler University of Linz (1002/2021).

## Results

### Patients and clinical characteristics

Lung tissue samples from 92 patients and their clinical records were analyzed. There were 35 females (38%) and 57 males (62%). Median age was 78 years (range; 48–98 years). Males were significantly younger with a median age of 75 years (range; 48–95 years) compared to females with a median age of 83 years (range; 53–98 years; *p*=0.018). CT values at the time of diagnosis ranged from 12.5 to 38.2 (median, 22.9). Median hospitalization time was 9 days (range; 1–43 days). The longest stay at the ICU was 30 days (see Table 1). Younger age was associated with longer hospitalization times including intensive care unit (*p*=0.026 and *p*<0.001). Five (5%) patients received no particular oxygenation, 57 (62%) patients received non-invasive oxygenation, and 30 (32.7%) patients received invasive oxygenation including 11 (12%) patients who were treated with ECMO. Median time for ECMO treatment was 13 days (range; 1–24).

**Table 1:** DAD patterns and correlation

Parameter	Total	DAD pattern			*p*-value
		Predominant exudative	Mixed	Predominant proliferative	
Gender (female/male; *n*)	57/35	12/16	8/25	15/16	0.114
Age (years; median)	80 (48–98)	84 (66–92)	83 (48–95)	73 (50–98)	0.004
Initial CT value (median)	22.9 (12.5–38.12)	20.8 (13.8–30.5)	18.9 (12.5–33.2)	24 (12.6–38.2)	0.027
Hospitalization total (days; median)	9 (1–43)	7 (1–20)	8 (2–34)	13 (1–43)	0.274
Hospitalization ICU (days; median)	0 (0–30)	0 (0–13)	0 (0–19)	7 (0–30)	0.001

Only three patients showed no comorbidities as extracted from the medical records. Twenty-nine (31.5%) patients had one risk factor. Sixty (65%) patients had two or more risk factors.

Positive blood cultures during the hospitalization were recorded in 29 (32%) patients. Bronchioloalveolar lavages positive for pathogens were recorded in 18 (20%) cases.

### Histological findings

**Fig. 2:**
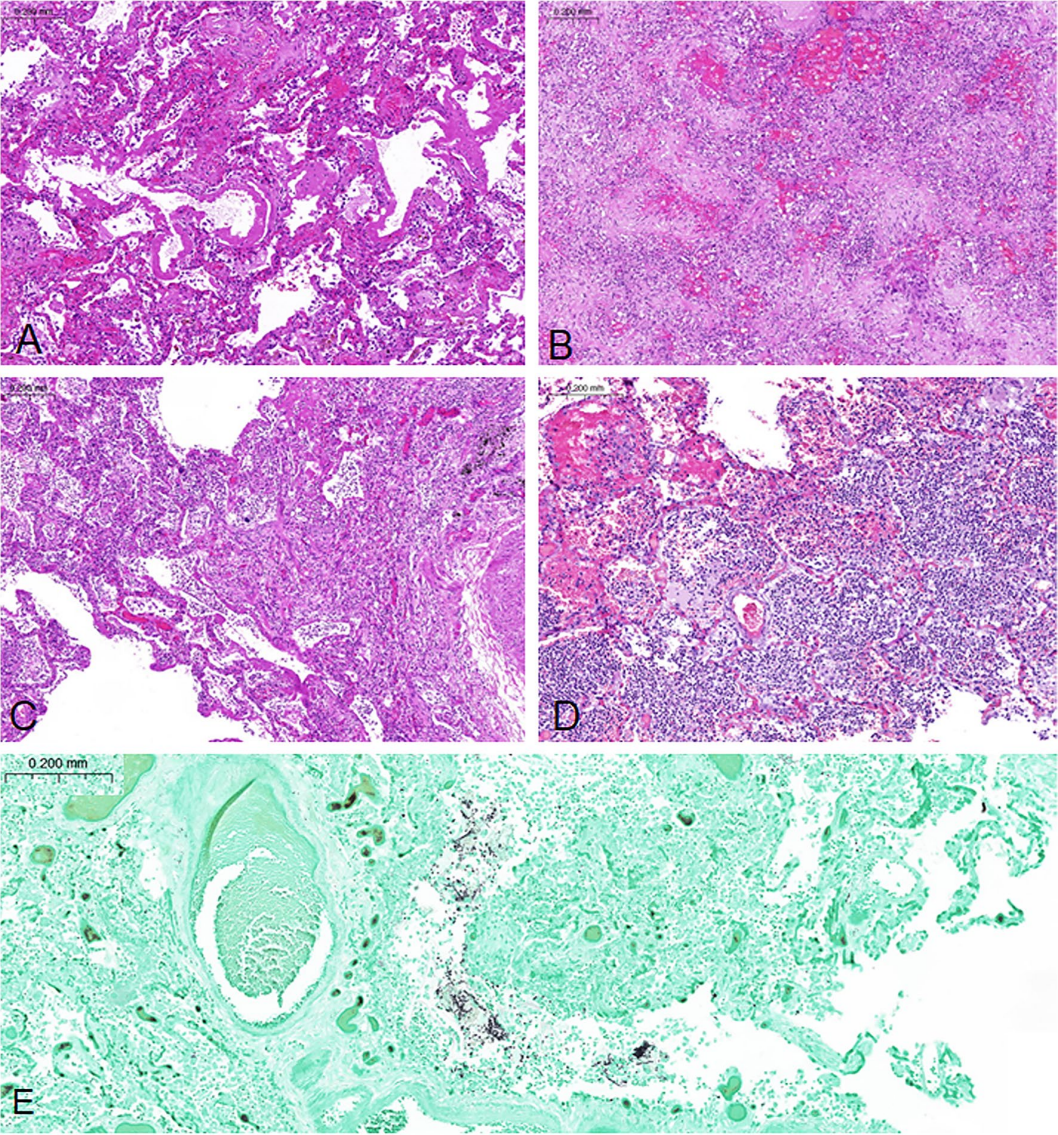
Histology. **A** DAD exudative phase; **B** proliferative phase; **C** mixed exudative and proliferative phase; **D** superimposed acute bronchopneumonia (all hematoxylin-eosin stain); **E** detection of fungal elements within areas of acute bronchopneumonia (Gomori-Grocott methenamine silver stain)

**Fig. 3:**
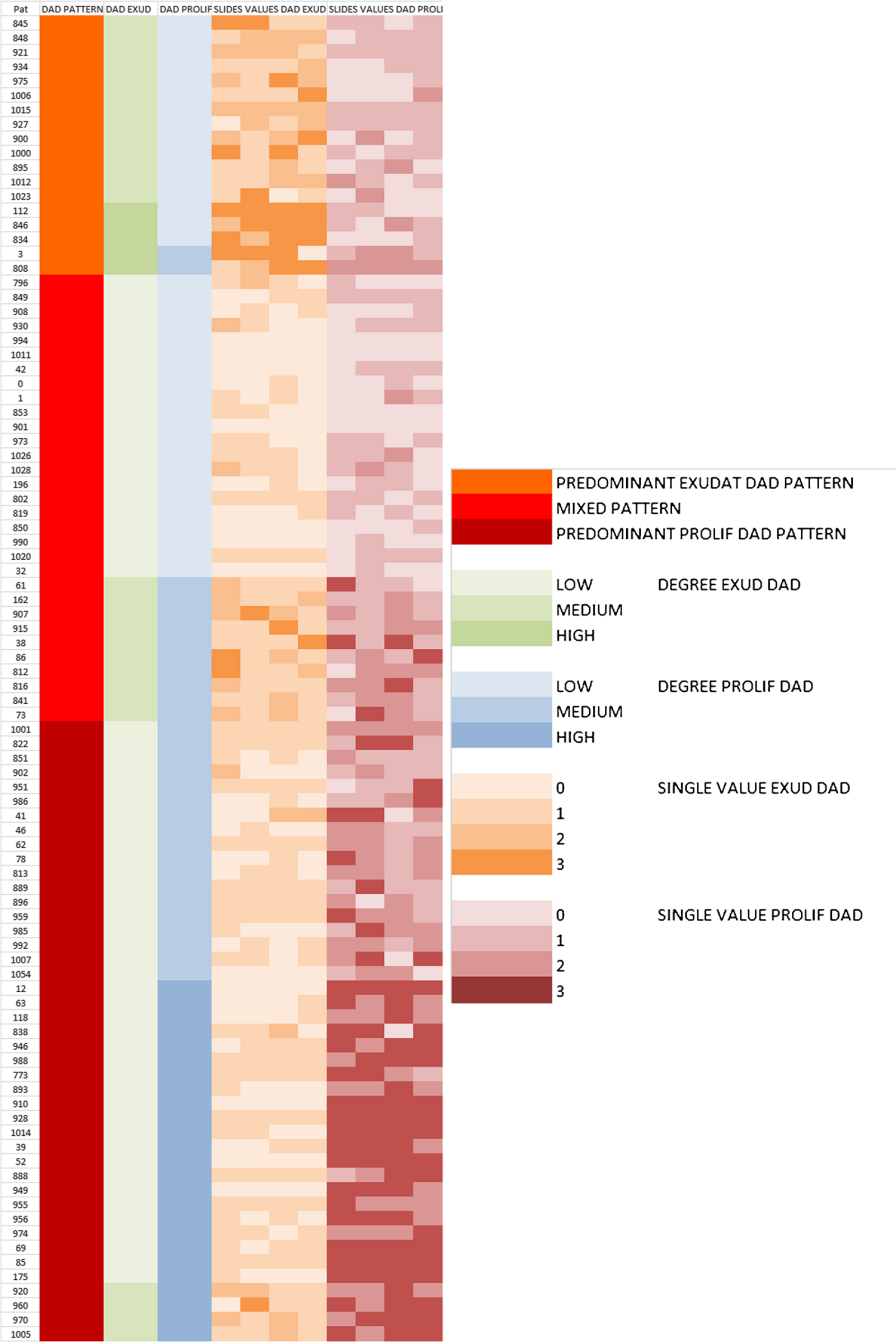
Illustration of DAD patterns and correlation with single values of degrees

DAD patterns were observed in various degrees, ranging from absent (score 0) to extensive (score 3). However, DAD (either eDAD or pDAD) was present in at least one slide in 89/92 (97%) patients. Pairwise comparison of the various categories of eDAD and pDAD across the slides revealed significant correlations between every pair (*p*<0.01 each). Deviations of more than one category in more than one slide were observed in three cases each for eDAD and pDAD (Figs. 2 and 3). After calculation of sum scores, the following categories were observed: eDAD was low in 60 (65%) patients, medium in 27 (29%) patients, and high in 5 (5%) patients. pDAD was low in 37 (40%) patients, medium in 30 (33%) patients, and high in 25 (27%) patients (Table 2). Predominant eDAD was seen in 18 (20%) patients, mixed pattern in 31 (34%) patients, and predominant pDAD was seen in 43 (47%) patients.

**Table 2:** DAD patterns and clinicopathologic parameters

		DAD proliferative			Total
		Low	Medium	High	
DAD exudative	Low	21	18	21	60
	Medium	13	10	4	27
	High	3	2	0	5
Total		37	30	25	92

Unequivocal microthrombi were not seen in 55 (60%) cases, low amounts were detected in 19 (21%) cases, and high amounts in 18 (20%) cases. Macrothrombi were not detected in 70 (76%) patients, and were detected in low amounts in 16 (17%) patients, and in high amounts in 6 (7%) patients. Squamous metaplasia was not detected in 28 (30%) patients and was present in low amounts in 20 (22%) patients and in high amounts in 44 (48%) patients. Superimposed bronchopneumonia was detected in 46 (50%) patients, including 22 (24%) with high level.

Squamous metaplasia correlated positively with the degree of pDAD (*p*=0.005), but not with the degree of eDAD (*p*=0.272), as well as with DAD patterns: high amounts of squamous metaplasia occurred more frequently in predominant pDAD patterns compared to predominant eDAD and mixed patterns. High amounts of microthrombi were more frequently seen in high level eDAD (*p*<0.001), but not in high level pDAD (*p*=0.24) and more frequently in predominant eDAD patterns compared to mixed and predominant pDAD (*p*<0.001). Macrothrombi were not significantly associated with degrees or patterns of DAD. Presence of bronchopneumonia as well as fungi occurred in both eDAD and pDAD without significant correlations with degrees or patterns. Of note, however, all cases with detection of fungi showed superimposed bronchopneumonia irrespective of the degree of inflammation (*p*=0.002).

### Correlation between histological and clinical findings

Patients with mixed DAD patterns had initially lower CT values (*p*=0.049). Younger age was associated with a predominant DAD proliferative pattern (*p*=0.019) but this was also linked to longer hospitalization times including intensive care unit (*p*=0.026 and *p*<0.001, Table 1). Moreover, younger patients received more frequently invasive oxygenation (*p*<0.001). On the other hand, pDAD pattern itself and higher degrees of pDAD were seen more frequently in association with invasive oxygenation methods which in turn were associated with longer stay at the ICU (*p*<0.001) but not statistically significant with the duration of hospitalization itself (*p*=0.289).

Positive results from bronchioalveolar lavages and blood cultures were more frequently seen in high degrees of pDAD (*p*<0.001; *p*=0.037) and in predominant pDAD patterns compared to predominant eDAD or mixed (*p*<0.001; *p*=0.018). Interestingly, there was no significant association between intravital positive microbiological results and the detection of superimposed bronchopneumonia or fungi at autopsy.

## Discussion

We report our experiences of the application of a simple intercostal minimally invasive autopsy approach for the collection and analysis of lung tissue of patients who died of COVID-19 during the European second wave of the disease between October 2020 and April 2021. For histological analysis, we obtained four tissue samples per patient measuring 5–8 cm. Almost all patients showed DAD in various stages, including exudative stage of DAD, proliferating stage of DAD, or mixed patterns. As expected, the proliferating pattern was more frequently observed in patients with longer total and intensive care unit hospitalization. The correlation between younger age and duration of the hospitalization until death, which may be due to better medical conditions in younger patients, may explain the association between age and DAD pattern. Interestingly, in almost half of the patients, superimposed acute bronchopneumonia was observed and fungi were detected in a small number of patients.

MIA procedures have been successfully introduced in post mortem diagnostic practices [28, 29] and suggested a valuable tool in particular situations as low resource settings, investigation of infectious diseases, and in cases where ethical, religious, or other restraints may hamper the consent for autopsy [24, 30, 31]. Often, the application of core needle biopsies is described, also in the context of COVID-19 [32, 33]. This approach, especially with ultrasound guidance, has the advantage of targeted sampling but requires expertise in imaging and the availability of imaging devices. With this technique, the amounts of harvested tissue are rather low in comparison to conventional post mortem histology sampling. Other techniques such as thoracoscopic or laparoscopic approaches also depend on technical equipment and expertise and may not be suitable for specific situations with acute or chronic inflammatory changes such as adhesions and fibrosis [28, 34]. Of note, a consent terminology that more precisely describes the different MIA techniques is still lacking. The term MIA should be therefore considered an umbrella for different methods ranging from rather simple approaches to highly sophisticated and technically demanding techniques. Our minimally invasive approach for the investigation of lung tissue with the creation of a small intercostal window and subsequent manual preparation would represent a rather simple technique. However, it delivers larger tissue samples which allow even a macroscopic evaluation of the obtained tissue followed by selective embedding for histology. It can be performed within a short time frame without requirement of additional technical devices. In theory, this approach may also allow a piecemeal preparation of the complete organ. In addition, the expansion of this concept to other organs such as the heart, liver, or kidneys is possible and is now sometimes used in our practice on request but currently without further scientific evaluation.

Despite the concept of COVID-19 being a multiorgan involvement of this viral disease [9], the main clinical focus lies on the lungs with respiratory failure as the most frequent reason for hospitalization including submission to the hospital or intensive care unit. Lung damage can be found in the vast majority if not in all patients [6, 11]. In our study, we therefore focused on pulmonary pathology.

On histopathological evaluation, our findings completely confirm what could be expected from early data published in case series [2–4, 10, 14, 26] and comprehensive review articles [9–11] and also what we had observed in our routine autopsy: almost all patients showed lung damage with the pattern of diffuse alveolar damage in various stages ranging from the exudative phase to proliferative phase including a considerable number of patients with mixed patterns. These findings are also in line with a recent publication [14] comprising a similar number of cases, in which DAD was observed in 82 of 100 patients, early/exudative DAD in 54 patients, and organization/proliferative DAD in 28. Microthrombi, which have been reported to occur frequently in SARS-COV-2-induced DAD [2, 3] and DAD of other etiologies [35], were detected more frequently in predominant exudative stages and 40% of patients. Of note, the frequency of this finding was lower than in initial reports [36] including our own observations [26]. This is also true for macrothrombi where frequencies up to 40% have been described in autopsies of the first wave of the pandemic [3, 26, 37]. The lower frequency found in our study may be explained by the later introduction of intensified systematic anticoagulant therapy soon after the publication of the first autopsy studies that highlighted this high frequency of thromboembolic events in COVID-19 [38].

Features indicative of the fibrotic phase of diffuse alveolar damage, such as mural fibrosis and microcystic honeycombing, were not observed. This is in contrast to recent publications suggesting that a progress towards fibrotic DAD occurs also in patients who died due to COVID-19 [39, 40] but concordant with the finding of Wu et al. [8] or Bryce et al. [14], where none of the investigated patients reached fibrotic stage. In this study, the median duration between the onset of symptoms and death was 11 days for early and 26 days for organizing DAD, which may be too short to establish complete fibrotic transformation. Carsana et al. hypothesized that pre-existing chronic and fibrotic lung damage may exacerbate by viral tissue toxicity. We did not observe a particular trend for fibrotic changes in the patient population with pulmonary comorbidities as documented in the medical files. Advanced fibrotic changes were observed to be focal, suggesting that none of the patients had completely progressed to the fibrotic phase of DAD, possibly because of the short duration of the disease [7].

Of note, marked heterogeneity in severity of the different DAD patterns seemed not to be an issue, as highly significant correlations between the main tissue findings of the four samples of each patient were observed and major deviations within the respective patterns occurred only in few cases. However, the number of tissue sections obtained was limited and sampling was not standardized in terms of precise anatomic allocation.

Another interesting and clinically relevant finding was the presence of superimposed acute bronchopneumonia which was observed in around half of the patients. Similar findings were reported before [6, 7, 26, 41]. This complication represents rather a true bacterial superinfection than an aggravating course of the viral disease. In our study, intravital laboratory findings of blood cultures and/or bronchioalveolar lavages did not correlate with histology. We could therefore not prove or contradict a bacterial superinfection in our post mortem examination in all cases, which may also be due to prior antimicrobial treatment before death. Empiric broad-spectrum antibacterial treatment may often be administered in patients with critical disease progression, even in the absence of a prove of bacterial co-infection [42, 43]. Rates of secondary pulmonary infections seem to be rather low in hospitalized COVID-19 patients with around 16% for bacterial infections and 6% for fungal infections, respectively [44]. However, the risk of co-infections increases with the severity of the disease with early admission to ICU, respiratory failure, corticosteroid therapy, and the length of hospitalization [45]. In our study, positive microbiological findings during hospitalization, however, were identified more frequently in patients with predominantly proliferative DAD pattern but this may also be linked to the longer hospitalization and associated risk of superinfection.

Especially fungal superinfections due to Aspergillus, Mucorales, or Candida species result in high mortality rates of more than 50% in critically ill COVID-19 patients [46, 47]. The absence of autopsies may also have resulted in an underestimation of the prevalence of fungal superinfections at the early phase of the COVID-19 pandemic, while diagnostic tests show a poor performance and consensus definitions were lacking [46, 47]. Also, in line with previous reports, we could demonstrate the presence of fungal elements in a small but considerable number of patients [48]. Histologically, it was also associated with a suppurative component but not with other pathological or clinical parameters. Similar to bacterial superinfection, this also includes the lack of a correlation with the findings of intravital microbiologic tests. Interestingly, we did not observe the typical image of invasive aspergillosis as observed in our own previous work on autopsy cases of the first wave [26] and anecdotally in patients who were autopsied in clinical routine setting after the study in our institute. Although the histological findings were rather similar in all four samples submitted for histology, we cannot exclude a sampling bias. COVID-associated pulmonary aspergillosis, with a reported incidence of up to 20%, has been reported a major clinical problem in the management of SARS COV-2 infections [49–52]. For autopsies in the context of this particular clinical setting, it may therefore be useful to consider prior imaging for a more targeted and extensive sampling in case of MIA or the investigation of the complete organ despite safety and organization issues.

In summary, we could observe the characteristic lung findings of DAD in exudative or proliferative stages, or of mixed pattern without significant heterogeneity of the DAD patterns in this unselected and unbiased longitudinal one center study of patients who died due to COVID-19 during the second wave of the pandemic between October 2020 and April 2020. Around half of the patients showed additional suppurative bronchopneumonia, with the detection of fungal elements in a few cases. Of note, although positive results of microbiological test were more frequently observed in patients with longer hospitalization, there was no association between microbiological test results and histological DAD patterns or the presence of a suppurative component. Further investigation of these discrepancies may have an impact on clinical management and surveillance of hospitalized COVID-19 patients. Besides adding further morphology-based evidence to the body of knowledge about COVID-19, our minimally invasive approach was proven to work in clinical practice and seemed to deliver reliable information because our findings did not differ significantly from data published from other authors so far. A more detailed evaluation of its reliability by standardized comparison with full autopsies, which could not be performed during the introduction due to the abovementioned infectious risk situations, would be valuable as the concept may be expanded to the investigation of other organ systems and can be proposed as a feasible approach in particular for autopsies in an infectious setting.
